# Supplementary material for: Ligament injury in adult zebrafish triggers ECM remodeling and cell dedifferentiation for scar-free regeneration
Source: NPJ Regen Med. 2023 Sep 19;8:51. doi: 10.1038/s41536-023-00329-9 (PMC10509200; doi:10.1038/s41536-023-00329-9)
Supplement: Supplementary file 1 — Supplementary Information [file 41536_2023_329_MOESM1_ESM.pdf]

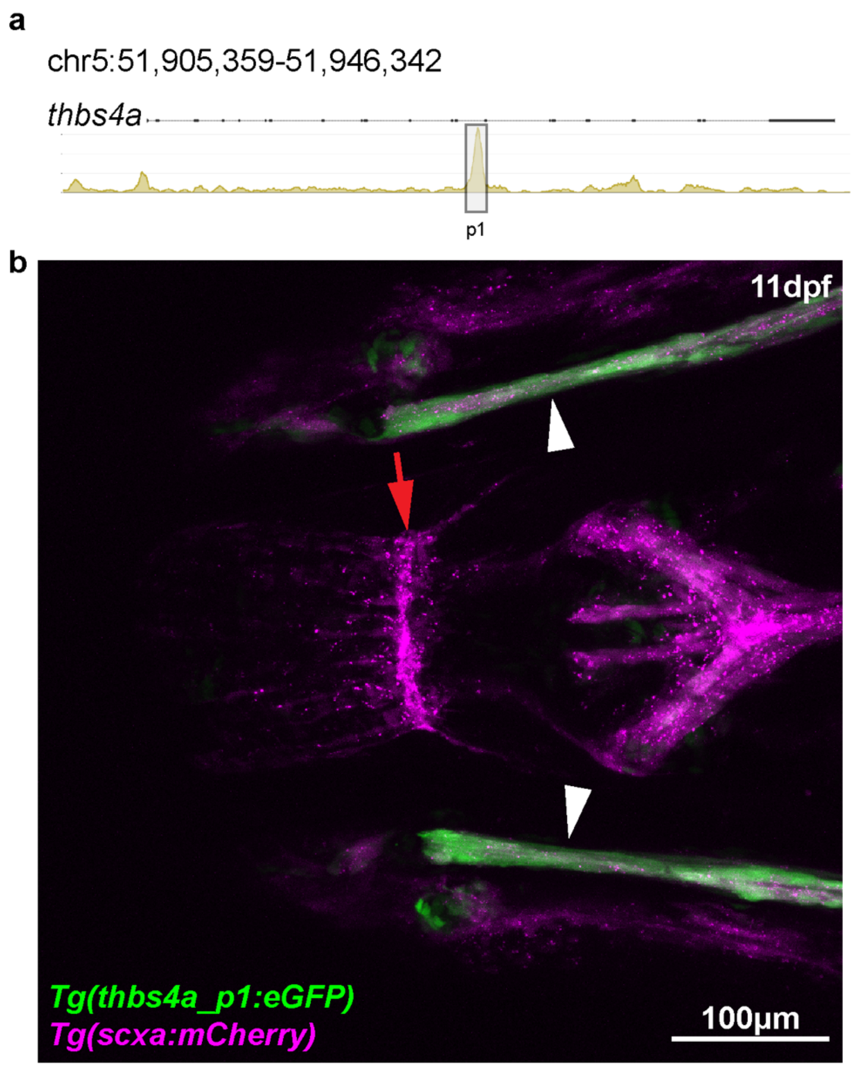

Supplementary Figure 1. Identification of *thbs4a_p1* enhancer and the developmental expression of *thbs4a_p1:eGFP* and *scxa:mCherry* in 11dpf embryos.

(**a**) Zebrafish *thbs4a* locus within Chromosome 5 with scATACseq track of chromatin accessibility in cranial neural crest-lineage ligament/tendon cell cluster shown below in gold. The *thbs4a_p1* intronic enhancer sequence location and increased ATAC accessibility is outlined in the p1 box. (**b**) Representative images of WT transgenic *Tg(thbs4a_p1:eGFP/scxa:mCherry)* zebrafish at 11 days post fertilization (dpf). Red arrows denote the midline tendon that is single positive for *scxa:mCherry* expression. White arrowheads denote double positive

thbs4a_p1:eGFP/scxa:mCherry signal in the bilateral IOM ligaments. *thbs4a_p1:eGFP* expression in the craniofacial region is limited to ligament cells and articular cartilage in the developing lower jaw joint (n=3). Scale bar= 100 μm.

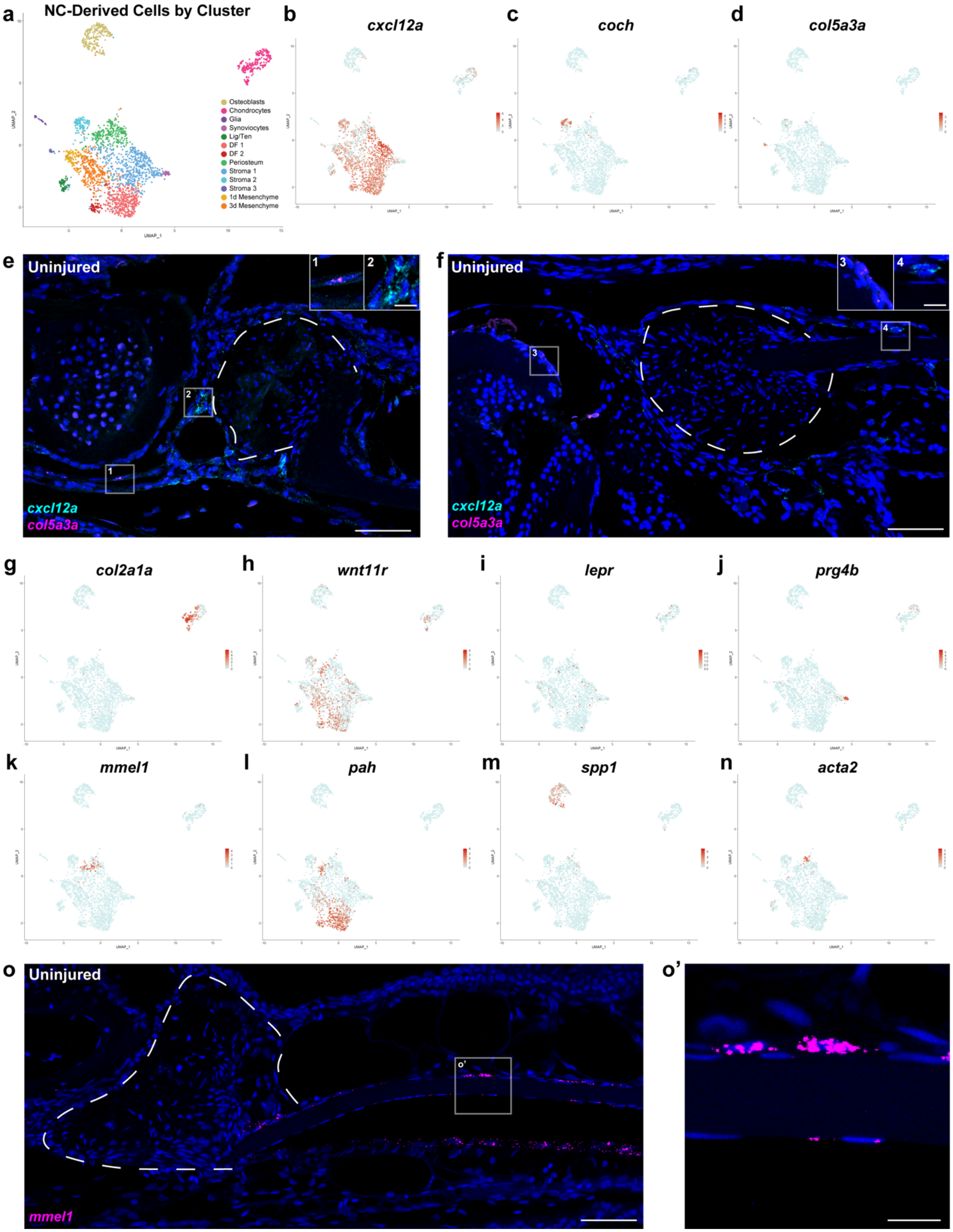
a
NC-Derived Cells by Cluster
Osteoblasts
Chondrocytes
Glia
Synoviocytes
Lig/Ten
DF 1
DF 2
Periosteum
Stroma 1
Stroma 2
Stroma 3
1d Mesenchyme
3d Mesenchyme
b
cxcl12a
c
coch
d
col5a3a
e
Uninjured
cxcl12a
col5a3a
f
Uninjured
cxcl12a
col5a3a
g
col2a1a
h
wnt11r
i
lepr
j
prg4b
k
mmel1
l
pah
m
spp1
n
acta2
o
Uninjured
mmel1
o'

Supplementary Figure 2. scRNAseq and smFISH characterization of tissue heterogeneity in adult zebrafish joints.

(a) UMAP illustrating clustering of neural crest-derived cells in early ligament regeneration. **(b-d)** FeaturePlots representing the expression of markers for clusters Stroma 1 (*cxcl12a*), Stroma 2 (*coch*), and Stroma 3 (*col5a3*). **(e-f)** RNAscope smFISH for *cxcl12a* (cyan) and *col5a3* (magenta) near uninjured ligament (white dashed line) (n=3). **(g-j)** FeaturePlots showing the expression of published markers of deep (*col2a1a*, *wnt11r*), intermediate (*lepr*), and shallow (*prg4b*) articular chondrocytes. **(k-n)** FeaturePlots illustrating markers of subsets of the Periosteum cluster, containing subsets of periosteum (*mmel1*) resembling dermal fibroblasts (*pah*) or pre-osteoblasts (*spp1*), as well as perivascular cells (*acta2*). **(o-o')** RNAscope smFISH demonstrating *mmel1* transcripts (magenta) in periosteal cells lining the IOP bone (n=3). Scale bar = 50 µm (e,f,o), 10 µm (o').

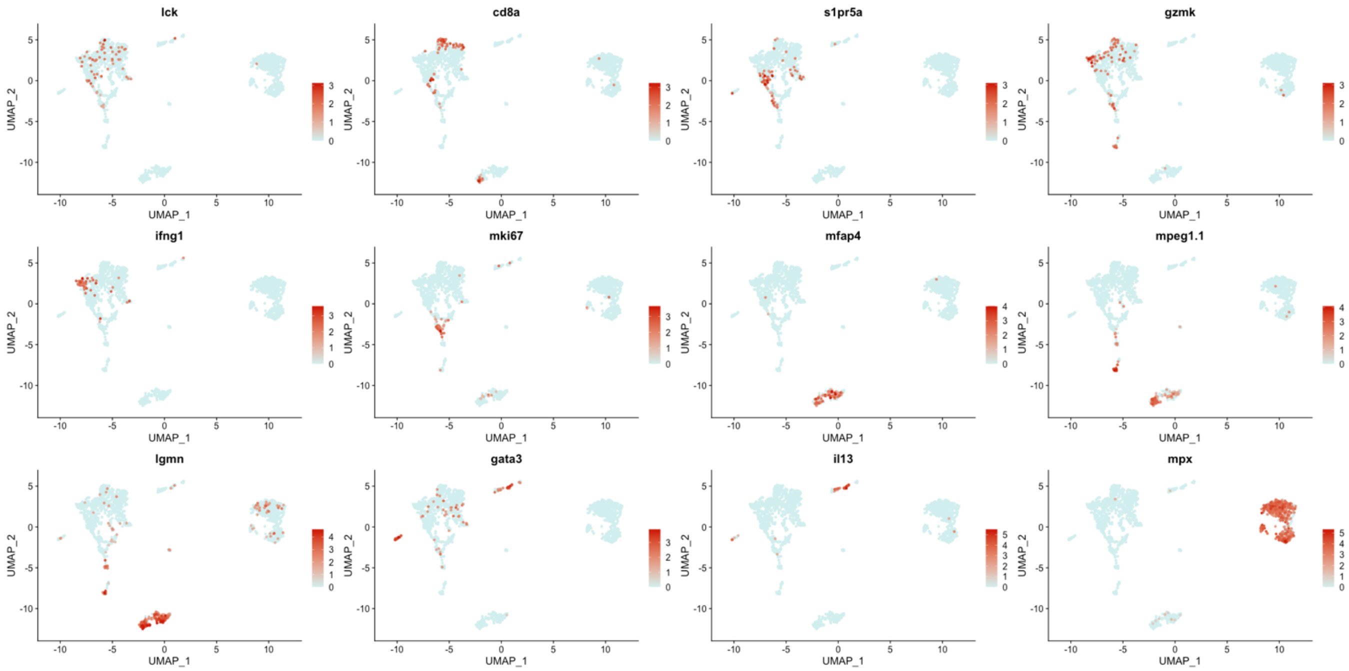

Supplementary Figure 3. scRNAseq feature plot expression for cluster-defining genes in immune populations.

Feature plots of mRNA expression of immune cluster marker genes.

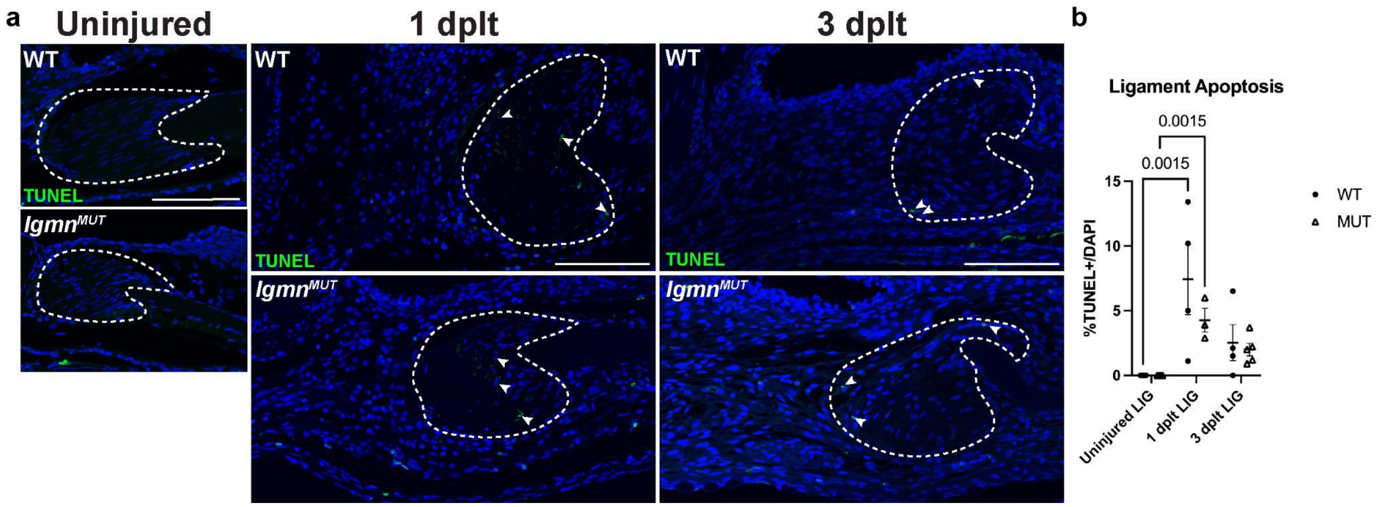

Supplementary Figure 4. Apoptosis of ligament cells following transection injury.

(a) Representative images of TUNEL staining in uninjured, 1 dplt, 3 dplt wildtype and *lgmn* mutants. Ligament region of interest for quantification is outlined in white dashed lines. Representative TUNEL positive nuclei indicated by white arrowheads. **(b)** Quantification of TUNEL positive ligament cells as a percentage of total DAPI+ nuclei in the region of interest shows increased ligament cell apoptosis in both wildtype and *lgmn* mutants at 1 dplt (n=3-5 per time point per genotype). Scale bars= 100 µm.

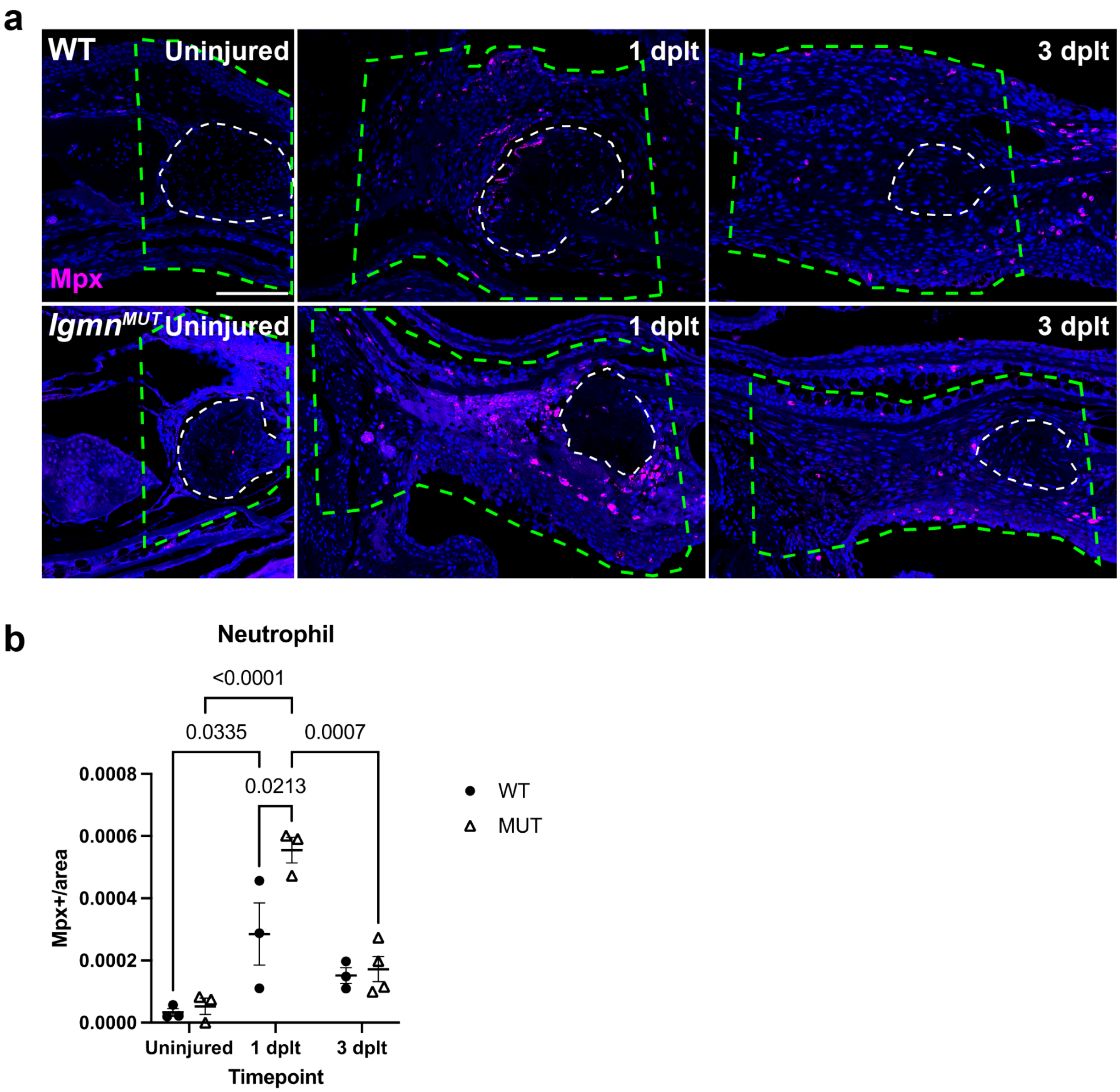

Supplementary Figure 5. *lgmn* mutants have elevated levels of neutrophils at the injury site at 1 dplt. (a) Representative images of immunofluorescence staining using Mpx antibody (magenta) in IOM ligaments in WT and *lgmn* mutants from uninjured, 1 dplt, and 3dplt (scale bar: 100μm). **(b)** Blinded quantification of Mpx+ cells/injury area. Region of interest for quantification is outlined in a green dashed line and the IOM ligament is outlined in a white dashed line. (n=3 per time point).

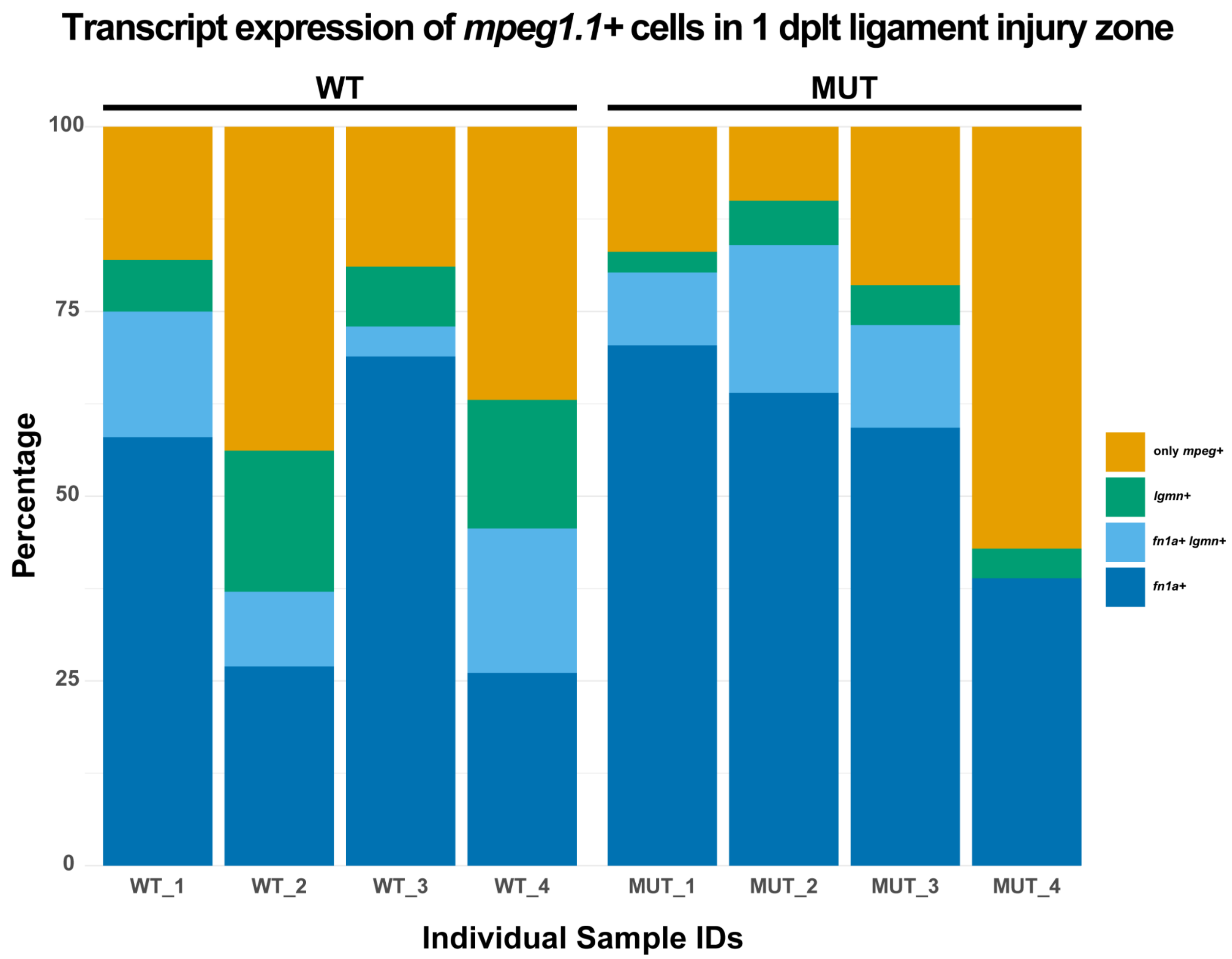

Supplementary Figure 6. Distribution of macrophage expression of *fn1a* and *lgmn* by sample ID at 1 dplt.

Stacked bar graphs showing the proportion of macrophages in individual biological replicate samples that are *mpeg1.1+* (yellow), *mpeg1.1+/lgmn+* (green), *mpeg1.1+/lgmn+/fn1a+* (light blue), or *mpeg1.1+/fn1a+* (dark blue) in wild type and *lgmn* mutants (n=4 per genotype).

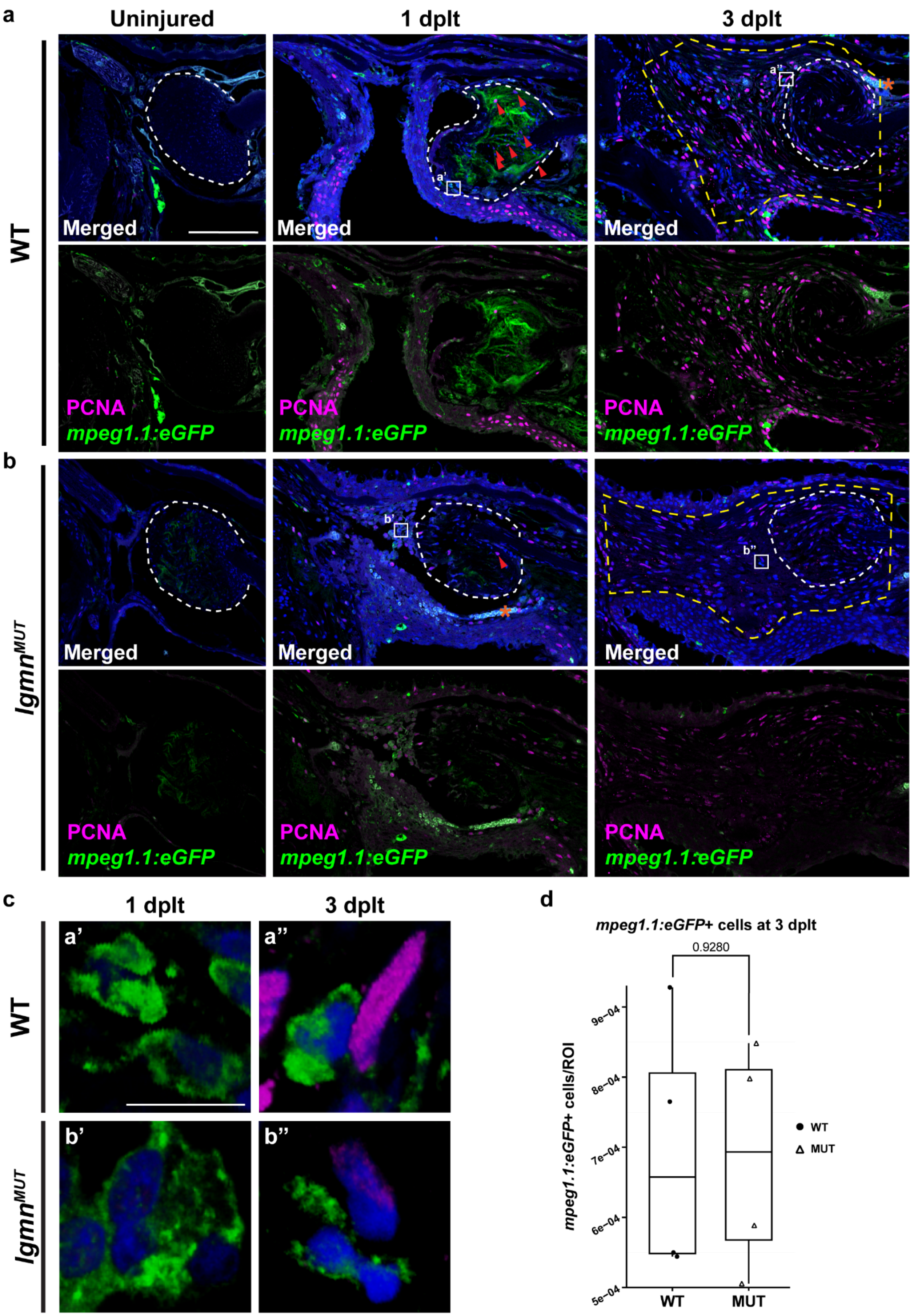
a
Uninjured
1 dplt
3 dplt
WT
Merged
PCNA
mpeg1.1:eGFP
a'
a''
b
lgmn^MUT
b'
b''
c
1 dplt
3 dplt
d
mpeg1.1:eGFP+ cells at 3 dplt
0.9280
mpeg1.1:eGFP+ cells/ROI
9e−04
8e−04
7e−04
6e−04
5e−04
WT
MUT

Supplementary Figure 7. Macrophage crosstalk with fibroblasts to promote proliferation.

(a-b) Representative images of eGFP and PCNA immunofluorescence staining of *Tg(mpeg1.1:eGFP)* uninjured, 1 dplt, and 3 dplt wild type and *lgmn* mutant IOM ligaments (a',b') *mpeg1.1:eGFP+* (green) cells not in contact with proliferating cells (magenta) at the transection site at 1 dplt. Red arrowhead denotes proliferating cells without coincident macrophages in the IOM ligament. Green staining in the IOM ligament seen in wild type 1 dplt and *lgmn* mutant uninjured are non-specific and are an artifact of the assay. (a", b") *mpeg1.1:eGFP+* cells in contact with proliferating cells near the transection site at 3 dplt **(c)** 3D renderings of 63x objective z-stacks of a'-a" and b'-b" regions outlined above. **(d)** Quantification of *mpeg1.1:eGFP+* cells/ROI in the ligament and mesenchyme domain at 3 dplt. IOM ligament is outlined in a white dashed line. Region quantified is outlined with a yellow dashed line. Orange * marks autofluorescent blood vessels. Scale bar = 100 µm (a-b), 10 µm (c). (n=3-4 per genotype)

Supplementary Methods 1

thbs4a_p1 sequence
> NC_007116.7:51926588-51927222 Danio rerio strain Tuebingen chromosome 5, GRCz11 Primary Assembly

CAAATATATATAGAGGTATACTGTCTAAAATGTCACTGAATGGAGCAAACTCCATCAGCAGAAA
AGCGTTGCACTGTTTTAGACTTGCATACAGTGACATCTTTGTGTGCGGTTATGTCACAAGGTG
GCAGTGTTTCAGAAAAAAAGACTTCTGTGGAATAGTTTCCACATGCCAGCAGCTTTTGGCTTG
GAATCTCTGTGGAAATGTGGTCGCTCGAAGGAGCCATGACCAAGCTTTAAAGCATCTAATCAA
GTGTCTGGATTTCTGCCTTGATGAGCAGACCCTAAATGTCAAACAACATCTGGACTCGATTTTA
CACATTTCAGACTGGAAGGAATAATTGTGCATTTAAAAGAAAATGATCAGTGTGGTGCAGTAA
ATGCAGCTAATATCTTTACAGGTCACTTCAGTTTTACATGACCCGCGCAAACTAAAGAGCGCTC
TAATTTGAGCTTTTAAAACACAGTAGCTTCTGTTTTTCCTCATAAAAATGACCTAAGCTCTGGA
AAATCCAGATAGAAAACAGGCCCGAAGGGCAAAAAGTATATTTAAAACACATCACCCCATTTC
TATGAATAAAAATGTGTCTTTGGCCACTTAGCAGCCGGATGACACGTGAAATGTTACTGCAGC
A

Supplementary Videos 1-4. 3D rendering of tissue-cleared imaging of *thbs4a_p1*:eGFP in uninjured (Video 1), 1 dplt (Video 2), 7 dplt (Video 3), and 28 dplt (Video 4) IOM ligaments.

Supplementary Table 1. scRNAseq cell counts by sample

Supplementary Table 2. Marker genes of neural crest lineage scRNAseq clusters

Supplementary Table 3. Marker genes of immune scRNAseq clusters
