# Supplementary material for: Ligament injury in adult zebrafish triggers ECM remodeling and cell dedifferentiation for scar-free regeneration
Source: NPJ Regen Med. 2023 Sep 19;8:51. doi: 10.1038/s41536-023-00329-9 (PMC10509200; doi:10.1038/s41536-023-00329-9)
Supplement: Supplementary file 5 — Reporting Summary [file 41536_2023_329_MOESM5_ESM.pdf]

nature portfolio

Corresponding author(s): Joanna Smeeton

Last updated by author(s): Aug 15, 2023

Reporting Summary

Nature Portfolio wishes to improve the reproducibility of the work that we publish. This form provides structure for consistency and transparency in reporting. For further information on Nature Portfolio policies, see our Editorial Policies and the Editorial Policy Checklist.

Statistics

For all statistical analyses, confirm that the following items are present in the figure legend, table legend, main text, or Methods section.

n/a	Confirmed	
☐	☒	The exact sample size (*n*) for each experimental group/condition, given as a discrete number and unit of measurement
☐	☒	A statement on whether measurements were taken from distinct samples or whether the same sample was measured repeatedly
☐	☒	The statistical test(s) used AND whether they are one- or two-sided<br>*Only common tests should be described solely by name; describe more complex techniques in the Methods section.*
☐	☒	A description of all covariates tested
☐	☒	A description of any assumptions or corrections, such as tests of normality and adjustment for multiple comparisons
☐	☒	A full description of the statistical parameters including central tendency (e.g. means) or other basic estimates (e.g. regression coefficient) AND variation (e.g. standard deviation) or associated estimates of uncertainty (e.g. confidence intervals)
☐	☒	For null hypothesis testing, the test statistic (e.g. *F*, *t*, *r*) with confidence intervals, effect sizes, degrees of freedom and *P* value noted<br>*Give P values as exact values whenever suitable.*
☒	☐	For Bayesian analysis, information on the choice of priors and Markov chain Monte Carlo settings
☒	☐	For hierarchical and complex designs, identification of the appropriate level for tests and full reporting of outcomes
☒	☐	Estimates of effect sizes (e.g. Cohen's *d*, Pearson's *r*), indicating how they were calculated

Our web collection on statistics for biologists contains articles on many of the points above.

Software and code

Policy information about availability of computer code

Data collection	Live imaging was performed using a Leica M165FC stereo microscope with Leica K5 sCMOS camera and LASX software v3.7.4.23463. Fluorescence imaging was performed using a Leica SP8 confocal microscope with LASX software v3.5.7.23225.
Data analysis	All statistical analysis was carried out using GraphPad Prism 9. Analyses of scRNAseq libraries were performed using R version 4.0.5 in RStudio with Seurat version 4.0.2.

For manuscripts utilizing custom algorithms or software that are central to the research but not yet described in published literature, software must be made available to editors and reviewers. We strongly encourage code deposition in a community repository (e.g. GitHub). See the Nature Portfolio guidelines for submitting code & software for further information.

Data

Policy information about availability of data

All manuscripts must include a data availability statement. This statement should provide the following information, where applicable:

- Accession codes, unique identifiers, or web links for publicly available datasets
- A description of any restrictions on data availability
- For clinical datasets or third party data, please ensure that the statement adheres to our policy

Raw and processed scRNA sequencing files have been deposited in GEO and can be retrieved using accession number GSE224197. All other raw data, transgenic zebrafish lines, and/or materials from this study are available from the corresponding author (JS) upon request.

Research involving human participants, their data, or biological material

Policy information about studies with human participants or human data. See also policy information about sex, gender (identity/presentation), and sexual orientation and race, ethnicity and racism.

Reporting on sex and gender	*Use the terms sex (biological attribute) and gender (shaped by social and cultural circumstances) carefully in order to avoid confusing both terms. Indicate if findings apply to only one sex or gender; describe whether sex and gender were considered in study design; whether sex and/or gender was determined based on self-reporting or assigned and methods used. Provide in the source data disaggregated sex and gender data, where this information has been collected, and if consent has been obtained for sharing of individual-level data; provide overall numbers in this Reporting Summary. Please state if this information has not been collected. Report sex- and gender-based analyses where performed, justify reasons for lack of sex- and gender-based analysis.*
Reporting on race, ethnicity, or other socially relevant groupings	*Please specify the socially constructed or socially relevant categorization variable(s) used in your manuscript and explain why they were used. Please note that such variables should not be used as proxies for other socially constructed/relevant variables (for example, race or ethnicity should not be used as a proxy for socioeconomic status). Provide clear definitions of the relevant terms used, how they were provided (by the participants/respondents, the researchers, or third parties), and the method(s) used to classify people into the different categories (e.g. self-report, census or administrative data, social media data, etc.) Please provide details about how you controlled for confounding variables in your analyses.*
Population characteristics	*Describe the covariate-relevant population characteristics of the human research participants (e.g. age, genotypic information, past and current diagnosis and treatment categories). If you filled out the behavioural & social sciences study design questions and have nothing to add here, write "See above."*
Recruitment	*Describe how participants were recruited. Outline any potential self-selection bias or other biases that may be present and how these are likely to impact results.*
Ethics oversight	*Identify the organization(s) that approved the study protocol.*

Note that full information on the approval of the study protocol must also be provided in the manuscript.

Field-specific reporting

Please select the one below that is the best fit for your research. If you are not sure, read the appropriate sections before making your selection.

☒ Life sciences ☐ Behavioural & social sciences ☐ Ecological, evolutionary & environmental sciences

For a reference copy of the document with all sections, see nature.com/documents/nr-reporting-summary-flat.pdf

Life sciences study design

All studies must disclose on these points even when the disclosure is negative.

Sample size	Sample sizes were determined based on expected effect size of interest with a minimum of 3 biological replicates for any given experiment.
Data exclusions	No data were excluded.
Replication	At least two independent technical replicates in a minimum of 3 biological replicates per condition were performed for all experimental findings. Two independent libraries per timepoint were sequenced or the scRNAseq analysis.
Randomization	Age and size matched animals were randomly assigned to treatment groups.
Blinding	Investigators were blinded during the data analysis to the sample genotype and injury prior to quantification and statistical analysis.

Reporting for specific materials, systems and methods

We require information from authors about some types of materials, experimental systems and methods used in many studies. Here, indicate whether each material, system or method listed is relevant to your study. If you are not sure if a list item applies to your research, read the appropriate section before selecting a response.

Materials & experimental systems

n/a	Involved in the study
☐	☒ Antibodies
☒	☐ Eukaryotic cell lines
☒	☐ Palaeontology and archaeology
☐	☒ Animals and other organisms
☒	☐ Clinical data
☒	☐ Dual use research of concern
☒	☐ Plants

Methods

n/a	Involved in the study
☒	☐ ChIP-seq
☒	☐ Flow cytometry
☒	☐ MRI-based neuroimaging

Antibodies

Antibodies used

PCNA, Sigma-Aldrich, P8825, lot 0000161007
mCherry, Novus, NBP2-25157SS, lot 357-110922
Mpx, GeneTex, GTX128379, lot 4434
eGFP, OriGene, TP401, lot 081211

Validation

All antibody staining was validated for non-specific binding through no primary antibody controls.
PCNA - as noted on manufacturer page, this Ab has been used in at least 164 published manuscripts. https://www.sigmaaldrich.com/US/en/product/sigma/p8825
mCherry - as noted on manufacturer page, this Ab has been used in at least 11 publications for the same application. https://www.novusbio.com/products/mcherry-antibody_nbp2-25157
Mpx - this Ab was specifically designed for zebrafish Mpx reactivity and has been used in at least 20 publications. https://www.genetex.com/Product/Detail/Mpx-antibody/GTX128379
eGPF - manufacturer's page details specificity for GFP and eGFP variants and has been cited at least 20 times. https://www.origene.com/catalog/antibodies/primary-antibodies/tp401/gfp-rabbit-polyclonal-antibody

Animals and other research organisms

Policy information about studies involving animals; ARRIVE guidelines recommended for reporting animal research, and Sex and Gender in Research

Laboratory animals

Zebrafish lines used in this study include AB, Tübingen, lgmnsa22350, Tg(scxa:mCherry), Tg(actab2:loxP-BFP-STOP-loxP-dsRed)sd2752, Tg(Mmu.Sox10-Mmu.Fos:Cre), Tg(mpx:mCherry), Tg(mpeg1:EGFP), Tg(thbs4a_p1:CreERT2) and Tg(thbs4a_p1:eGFP). All surgical injuries were performed in size-matched 3-6 months post fertilization (mpf) adult zebrafish using standard body length (SL) measurements.

Wild animals

The study did not involve wild animals.

Reporting on sex

Male and female zebrafish were included in the study design as no sex-related differences in regeneration were noted. Each experiment has both sexes aggregated in each sample.

Field-collected samples

The study did not include field-collected samples.

Ethics oversight

The Institutional Animal Care and Use Committees of Columbia University and the University of Southern California approved all zebrafish experiments.

Note that full information on the approval of the study protocol must also be provided in the manuscript.
